# Comparison of statistical methods for the analysis of patient-reported outcomes in randomised controlled trials: A simulation study

**DOI:** 10.1177/09622802241275361

**Published:** 2024-10-23

**Authors:** Yirui Qian, Stephen J Walters, Richard M Jacques, Laura Flight

**Affiliations:** 1 Centre for Health Economics, University of York, York, UK; 2 Division of Population Health, School of Medicine & Population Health, University of Sheffield, Sheffield, UK

**Keywords:** Statistical methods, patient-reported outcomes, Tobit model, Short Form 6-Dimension, statistical analysis, Monte Carlo simulation

## Abstract

Patient-reported outcomes (PROs) that aim to measure patients’ subjective attitudes towards their health or health-related conditions in various fields have been increasingly used in randomised controlled trials (RCTs). PRO data is likely to be bounded, discrete, and skewed. Although various statistical methods are available for the analysis of PROs in RCT settings, there is no consensus on what statistical methods are the most appropriate for use. This study aims to use simulation methods to compare the performance (in terms of bias, empirical standard error, coverage of the confidence interval, Type I error, and power) of three different statistical methods, multiple linear regression (MLR), Tobit regression (Tobit), and median regression (Median), to estimate a range of predefined treatment effects for a PRO in a two-arm balanced RCT. We assumed there was an underlying latent continuous outcome that the PRO was measuring, but the actual scores observed were equally spaced and discrete. This study found that MLR was associated with little bias of the estimated treatment effect, small standard errors, and appropriate coverage of the confidence interval under most scenarios. Tobit performed worse than MLR for analysing PROs with a small number of levels, but it had better performance when analysing PROs with more discrete values. Median showed extremely large bias and errors, associated with low power and coverage for most scenarios especially when the number of possible discrete values was small. We recommend MLR as a simple and universal statistical method for the analysis of PROs in RCT settings.

## 1 Background

Patient-reported outcomes (PROs) are widely used to measure patients’ subjective attitudes towards their health or health-related conditions.^1,2^ PROs enable health researchers to measure, analyse and compare clinical outcomes from the patient's perspective, providing clinical effectiveness outcomes in clinical trials and evidence to support decision making in health technology assessment. There has been an overall increasing trend in using PROs as clinical outcomes in the United Kingdom's publicly funded randomised controlled trials (RCTs).^3^

RCTs are regarded as the gold standard for evaluating the effectiveness of health interventions.^4,5^ The randomisation process in a well-designed RCT can reduce selection and allocation bias, and inform the causality of the treatment on responses.^6^ These traits of RCTs can simplify the data analysis of PROs. PROs produce numerical scores and generate distributions that are typically bounded, discrete, and skewed, which complicates the decision on what statistical methods to use for analysis. An inappropriate analysis can result in unreliable estimates and fail to provide accurate and robust results for decision-making on the use of health interventions.

An estimand is a detailed description of what treatment effect a trial is trying to focus on. It illustrates what the numerical result found in the trial will represent.^7,8^ An estimand is composed of five connected attributes: the population, the treatments to compare, the outcome or endpoint, how to account for intercurrent events, and a population-level summary measure of how the outcome between the different treatment conditions will be compared.^9^

PROs in RCTs are frequently analysed with statistical methods that are part of the family of general linear models, particularly linear regression, analysis of covariance, and linear mixed models,^3^ where the population summary measure of how the outcome between the different randomised groups will be compared is the difference in group means. There are several alternative statistical methods to general linear models for analysing PRO, some of which have a similar estimand (e.g. bootstrapping,^10^ Tobit regression,^11^ and quantile regression,^12^ where the population-level summary measure is a difference in means or medians); while other models (e.g. ordinal logistic regression^13^ and beta-binomial regression^14^) have a different estimand (where the population-level summary measure is an odds ratio).

However, as each method has its own model assumptions, estimation procedures, and estimands, it is still unknown which method is the most appropriate to analyse PROs, particularly in RCT settings.

Existing guidelines that have been published by government organisations, such as the Food and Drug Administration (FDA),^15^ and academic groups such as the Standard Protocol Items: Recommendations for Interventional Trials-PRO extension (SPIRIT-PRO)^16^ and the Consolidated Standards of Reporting Trials Statement-PRO extension (CONSORT-PRO)^17^ to standardise the use of PROs, mainly focused on the reporting of PROs or on the process of PRO development such as what health dimensions to cover, what items to include, and how feasible, valid, and reliable the PROs are. In terms of the statistical analysis of PROs, they provide guidance on what components to report and consider such as the targeted dimensions, the specification of the primary endpoint, and the statistical approaches to dealing with the missing data, but no guidance on what specific statistical methods should be considered to analyse PROs under different scenarios are provided.

As the ‘truth’ is unknown for real-world datasets,^18^ simulation analysis is used as a common approach to the evaluation of different statistical methods under various predefined scenarios, through which, investigation on how close the estimates produced by these methods are to the predefined ‘truth’, and whether the performance of these methods remain robust when analysing different dimension scores of PROs and when model assumptions are violated can be carried out. The existing literature, especially simulation studies, mainly focus on the application of different statistical methods for analysing health utility scores,^11,19–24^ while there is a lack of research in statistical methods for analysing PRO dimension scores.

This study aims to apply simulation analysis for the evaluation of different statistical methods for estimating the treatment effect of latent continuous PRO dimension scores with equally spaced discrete scoring under a two-arm balanced RCT, considering multiple scenarios. Since we believe the PRO is an underlying continuous variable then, using the estimand framework, an appropriate population-level summary measure for the treatment effect is a difference in measures of central tendency such as means or medians between the randomised groups. Therefore, the statistical methods for comparison in this study are multiple linear regression (MLR), Tobit regression (Tobit), and Median regression (Median), which generate mean or median estimates. Other statistical methods, for analysing PROs such as ordinal logistic regression and beta-binomial regression, estimate odds ratios or differences in proportions as the population-level summary measure of the treatment effect, and are therefore a different estimand and outside the scope of this study.

The rest of the paper is composed of the design of the simulation analysis; the comparison of model performance of targeted statistical methods in various scenarios; and discussion and recommendations on what statistical methods to use for the analysis of PRO dimension scores in RCT settings.

## 2 Methods

This section describes the techniques to conduct simulation analysis for the comparison of the accuracy and robustness of three statistical methods, i.e., MLR, Tobit, and Median. Multiple scenarios for PRO data with 4, 10, and 26 possible discrete values or scores (i.e. levels) were simulated using a random-data generator from a Normal distribution, followed by discretisation techniques that rescored the Normally distributed data into different numbers of possible discrete values. The ‘ADEMP’ strategy proposed by Morris et al.,^25^ which abbreviates the initials of aims, data-generating mechanism (DGM), estimands, methods, and performance measure, was adapted to develop this section.

### 2.1 Aims

This study aimed to evaluate the ability of MLR, Tobit, and Median to estimate the predefined treatment effects of latent continuous PROs with equally spaced discrete scores, also called the predefined ‘truth’, under a range of scenarios in balanced two-arm RCT settings.

### 2.2 Data-generating mechanism

Multiple DGMs were proposed to ensure the coverage of different scenarios, by varying the number of observations (i.e. sample size) and predefined treatment effects, and by considering PRO scores with different number of possible discrete values, such that each DGM provides us with empirical results for a specific scenario.^25^

The Short Form 36 Health Survey (SF-36) was used as the representative of PROs in this study as it is one of the most used PROs in publicly funded RCTs in the UK.^3^ The SF-36 is a multidimensional PRO that consists of 36 items on different discretised scales, which generates eight health dimension scores that are composed of two or more exclusive items and one additional item that measures health transition compared to the past.^26^ Modifications of the original SF-36 are developed, and the original version was chosen as the reference to generate simulated datasets since it contains more variability in the number of possible values that a dimension score can have. Similar DGMs were used to construct simulated datasets for three dimensions with different discrete values, including role limitation-emotional (RE) (k=4) , bodily pain (BP) ( k=10 ), and mental health (MH) (k=26) , where *k* is the number of possible discrete values the corresponding dimension has. These three dimensions were chosen because they have the lowest, median, and highest number of possible discrete values out of the eight dimensions in SF-36.^27–29^ The possible discrete values of these three dimensions, ranging from 0 to 100, are shown in Table 1.

**Table 1.** Possible discrete values of the three dimensions in SF-36 (RE, BP, and MH).

Dimensions	Possible discrete values
RE ( k=4)	0, 33.3, 66.6, 100
BP ( k=10)	0, 11.1, 22.2, 33.3, 44.4, 55.6, 66.7, 77.8, 88.9, 100
MH ( k=26)	0, 4, 8, 12, 16, 20, 24, 28, 32, 36, 40, 44, 48, 52, 56, 60, 64, 68, 72, 76, 80, 84, 88, 92, 96, 100

k represents the number of possible discrete values of a dimension score. SF-36: Short Form 36 Health Survey; BP: bodily pain; MH: mental health; RE: role limitation – emotional.

The Normal distribution, denoted by $Normal(\mu ,\sigma^{2})$ , was used to generate the PRO scores using Monte Carlo methods as we believe that the underlying PRO score is a latent continuous variable with no boundaries. Random samples were generated using the mean (μ) of 50 and standard deviation (SD, σ ) of 22 for the base-case DGM, based on evidence from a trial assessing the effectiveness of acupuncture therapy^30^ where the average mean score of its primary endpoint, SF-36 BP scores, for the control group is 58, with an SD of 22.

We also adapted a variation of predefined treatment effect ( x ), i.e. the predefined ‘truth’, by adding different magnitudes of predefined values to the observations in the treatment group and then analysing simulated datasets using these three statistical methods to measure their ability to detect the predefined treatment effect. The values of 0, 0.2, 0.5, 0.8, and 1.0 of Cohen's effect size^31,32^ were used to simulate different magnitudes of the treatment effect, representing no effect, small effect, median effect, large effect, and very large effect. With the value of SD fixed at 22, the treatment effect ( x ) was set at 0, 4.4, 11, 17.6, and 22, respectively. Table 2 presents the parameter specification using the Normal distribution. The same set of parameters are used for all three levels (i.e. RE, BP, and MH).

**Table 2.** Parameter specification for five DGMs using Normal distribution.

	Normal distribution		Additional value added to	
	generator (control group)		treatment group	
			Treatment	Cohen's
DGM number	Mean ( μ )	SD ( σ )	effect ( x )	effect size
1	50	22	0.0	0.0
2			4.4	0.2
3			11.0	0.5
4			17.6	0.8
5			22.0	1.0

DGM: data-generating mechanism; SD: standard deviation.

An issue of generating data from the Normal distribution is that the simulated dimension scores may go beyond the boundaries of 0 and 100 for the SF-36 BP score. The following strategy was used to deal with this issue. Firstly, simulated scores exceeding the lower and upper bounds were rounded to the values at boundaries, i.e. 0 or 100, and secondly, the values on the continuous scale were discretised onto an equally spaced discrete scale between 0 and 100. The discretisation techniques were determined by the number of possible discrete values of the simulated dimension score (Table 3). For example, RE scores with four possible values will be discretised into 0, 33.3, 66.6, or 100. A simulated continuous score of 10.5 would be discretised onto a value of 0 under level 4, a value of 11.1 under level 10, and a value of 12 under level 26.

**Table 3.** Discretisation techniques for the three dimensions in SF-36 (RE, BP, MH).

RE ( k=4 )			BP ( k=10 )			MH ( k=26 )		
Possible	Discretisation		Possible	Discretisation		Possible	Discretisation	
scores	techniques		scores	techniques		scores	techniques	
0	( −∞ ,	16.65]	0	( −∞ ,	5.55]	0	( −∞ ,	2]	
33.3	(16.65,	49.95]	11.1	(5.55,	16.65]	4	(2,	6]	
66.6	(49.95,	83.25]	22.2	(16.65,	27.75]	8	(6,	10]	
100	(83.25,	+∞ )	33.3	(27.75,	38.85]	12	(10,	14]	
			44.4	(38.85,	49.95]	16	(14,	18]	
			55.6	(49.95,	61.05]	20	(18,	22]	
			66.7	(61.05,	72.15]	24	(22,	26]	
			77.8	(72.15,	83.25]	28	(26,	30]	
			88.9	(83.25,	94.35]	32	(30,	34]	
			100	(94.35,	+∞ )	36	(34,	38]	
						40	(38,	42]	
						44	(42,	46]	
						48	(46,	50]	
						52	(50,	54]	
						56	(54,	58]	
						60	(58,	62]	
						64	(62,	66]	
						68	(66,	70]	
						72	(70,	74]	
						76	(74,	78]	
						80	(78,	82]	
						84	(82,	86]	
						88	(86,	90]	
						92	(90,	94]	
						96	(94,	98]	
						100	(98,	+∞ )	

k represents the number of possible discrete values of a dimension score. BP: bodily pain; MH: mental health; RE: role limitation – emotional.

The number of observations, i.e. sample size, of each simulated dataset, was set at 100, 200, 400, 800, 1200, and 1600, using evidence from 114 identified trials that used PROs as primary clinical outcomes.^3^ The sample sizes of these trials ranged from 65 up to 7677, with the 5th, 50th, and 95th percentile of 102, 387, and 1084, respectively. Given the right-skewed distribution of their sample size, the sample sizes of 1600 and 100 were used as the maximum and the minimum number of observations in each simulated dataset, respectively. Assuming two balanced arms in an RCT, half of the sample was assigned to the treatment group and the other half to the control group for each simulated dataset.

The number of repetitions was set at 5000 for all scenarios, since with 5000 number of simulations, the Monte Carlo standard error^25^ of the estimate, such as the coverage of 95% confidence interval (CI) estimates, is approximately 0.003, leading to approximate 95% CI for the coverage of 0.944–0.956, which is believed as a sufficient level of precision for the coverage performance measure.

### 2.3 Estimands

For this simulation analysis of the treatment effect in a PRO score at a specific post-randomisation between two randomised groups, four of the elements of the estimands framework (i.e. population, treatments, outcomes, and how to account for intercurrent events) were unchanged, but the fifth the population-level summary measure was the treatment effect between two parallel treatment arms measured by the means for MLR and Tobit, and medians for Median, which was also broadly the same if we assume both means and medians are measures of location or central tendency and the summary measure is a difference in measures of location or central tendency.

### 2.4 Methods of analysis

In MLR, the relationship between the mean of each PRO score and the linear predictor is described using the following equation:

Y=Xβ+ε

where the Y is a vector of observed PRO scores, X is the design matrix that denotes multiple row vectors of independent variables such as baseline score and treatment group, and ε is the error term that captures the difference between the linear predictor and the independent variable, and it is assumed to follow a Normal distribution with the mean of 0.

Median is a special case of quantile regression when the quantile level is set at the 50th. It estimates the conditional median of the response variable and does not assume a particular parametric distribution for the response. Median has a similar equation as MLR, but it depicts the relationship between the median of dimension scores, denoted by $Q_{Y|X}(median)$ , and the linear predictor.

$$Q_{Y|X}(median)=X\beta_{median}$$

where the 50th conditional quantile of Y is given as a linear function given X .

Tobit regression is an extension of MLR that estimates the mean. It describes the relationship between the latent variable and the linear predictor. It is a case of censored regression that assumes the boundaries of the dependent variable are due to censoring, that is, the mean of latent PRO scores, denoted by $Y^{*}$ , can exceed the upper and lower boundaries, but they are not observable. The observed dependent variable of the censored regression is defined using the following equations:

$$Y=\{\begin{array}{cc} a, & Y*\leq a\\ Y*, & a<Y*<b\\ b, & Y*\geq b \end{array}$$

where *a* and *b* denotes the lower and upper bounds of the PRO score, respectively.

$$Y^{*}=X\beta +\varepsilon$$

where $Y^{*}$ denotes the latent variable of the PRO score, which satisfies the classical linear model assumptions; whereas Y , the censored outcome of the latent variable $Y^{*}$ , does not have a linear relationship with X and ε .

Since the DGMs were simulated under the RCT settings, no other ${\hat{x}}_{i}$ factors were introduced to influence the treatment effect. Therefore, these methods were applied to the simulated datasets by estimating the treatment effect without adjusting for covariates.

### 2.5 Performance measures

The main performance measures were bias, coverage of the 95% CI, empirical standard error (EmpSE), mean squared error (MSE), Type I error under the null hypothesis of no treatment effect (DGM 1), and power under the alternative hypothesis of a variety of predefined non-zero treatment effects (DGM 2–5). The estimated treatment effect from each statistical method is denoted using ${\hat{x}}_{i}$ , and the pre-specified treatment effect in terms of the difference in group means or difference in group medians is denoted using x . The mean of  (i.e. the estimate of *x* from the *i*th repetition) across repetitions is denoted using $\bar{x}$ .

**Bias** $\text{(}\delta_{x})$ is defined as the average difference between the estimated values, ${\hat{x}}_{i}$ and the predefined ‘truth’, x . Due to the discretisation techniques attached to the DGMs, the exact ‘truth’ can never be known, except when the predefined treatment effect is zero.

$$\delta_{x}=\frac{1}{n_{sim}}\overset{n_{sim}}{\underset{i=1}{\sum }}\;{\hat{x}}_{i}-x$$

where $n_{sim}$ represents the number of repetitions used, and $i=1,\ldots ,n_{sim}$ indexes the repetitions of the simulation.

**Empirical standard error (** $EmpSE_{x}$ ) is the precision measure of the estimated values to the average ( $\bar{x}$ ) for each method. The predefined ‘truth’ ( x ) is not required to generate this measure.

$$EmpSE_{x}=\sqrt{\frac{1}{n_{sim}-1}\overset{n_{sim}}{\underset{i=1}{\sum }}\;{\left({\hat{x}}_{i}-\bar{x}\right)}^{2}}$$

**Mean** **squared error (** $MSE_{x}$ **)** measures the difference between estimated values to predefined ‘truth’ ( x ), and it can be calculated as the sum of the squared bias and variance of ${\hat{x}}_{i}$ .

$$MSE_{x}=\frac{1}{n_{sim}}\overset{n_{sim}}{\underset{i=1}{\sum }}\;({\hat{x}}_{i}-x{)}^{2}={\delta_{x}}^{2}+EmpS{E_{x}}^{2}$$

**Coverage of CIs** is defined as the probability that a CI contains the predefined ‘truth’ ( x ). For a (1−α) CI, the coverage is expected to be exactly (1−α) of the intervals containing *x*, else it is regarded as under- or over-coverage.

$$Coverage_{x}=\frac{1}{n_{sim}}\overset{n_{sim}}{\underset{i=1}{\sum }}\;1({\hat{x}}_{low,\;i}\leq x\leq {\hat{x}}_{upp,\;i})$$

where ${\hat{x}}_{low,\;i}$ and ${\hat{x}}_{upp,\;i}$ the lower and upper bounds of the CI, and this term takes the value of 1 if *x* is within the interval or 0 otherwise.

**Type I error**, known as the ‘false positive rate’, is defined as the probability of falsely rejecting the true null hypothesis that x=0 , using an α significance level of 0.05. **Power**, known as the ‘true positive rate’, is defined as the probability of correctly rejecting the null hypothesis, when the alternative hypothesis is true.

$$Type\;I\;error\;or\;Power_{x}=\frac{1}{n_{sim}}\overset{n_{sim}}{\underset{i=1}{\sum }}\;1(p_{i}\leq \alpha )$$

where $p_{i}$ is the *p*-value returned by the *i*th repetition and the term $\left(p_{i}\leq \alpha \right)$ takes the value 1 if the *p*-value is less than or equal to α or 0 otherwise.

Exploratory analysis will be carried out, mainly by graphs for each DGM, estimand, number of observations, and statistical method.^25^ The statistical package Stata/MP 17.0 was used for simulation analysis and MATLAB R2023a was used for data visualisation. The seeds and streams for the random-number generator were set the same in Stata to ensure that the exact same set of Normal distributions were used to produce scores for these three dimensions.

## 3 Results

This section reports the characteristics of the simulated datasets and evaluates the performance of three statistical methods (i.e. MLR, Tobit, and Median) for analysing latent continuous PROs with equally based scores in balanced two-arm RCT settings under multiple scenarios.

### 3.1 Characteristics of the simulated datasets

Following the five proposed DGMs, each simulation generated one simulated dataset assuming an underlying latent Normal distribution to randomly generate the outcome. The base case (DGM 1) has a mean of 50 and SD of 22. The simulated latent Normally distributed scores were then ‘discretised’ into an outcome with a discrete number of levels or scores (e.g. 4, 10, and 26 levels). Under each level, the mean PRO score in the control group was the same for all DGMs, but the mean PRO score in the treatment group varied by the five predefined treatment effects. With 5000 simulations and six sets of sample sizes (i.e. 100, 200, 400, 800, 1200, and 1600) per simulation, a total number of 30,000 simulated datasets (= 5000 simulations × 6 sets of sample sizes) were produced under each level, generating 2,700,000 estimates in total (= 30,000 simulated datasets × 6 methods × 5 DGMs × 3 levels).

**Figure 1.**
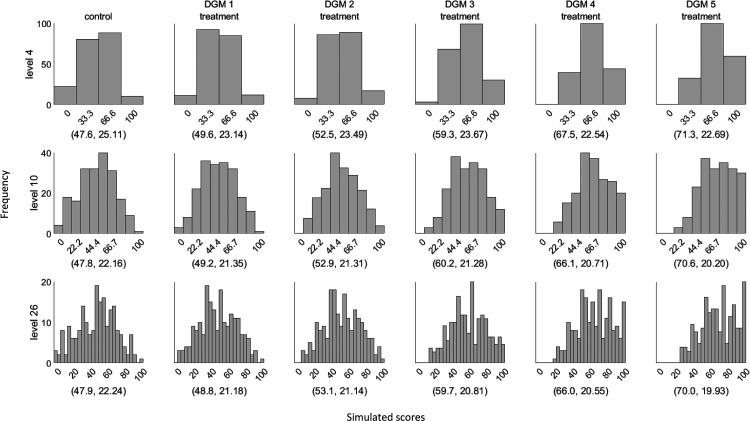
Example distributions of the simulated dataset using the five data-generating mechanisms (DGMs) for three different levels (sample size = 400). The values in the bracket represent the mean and standard deviation of the displayed distribution. The first column represents the score distribution of the control group. DGM 1–5 represents the score distributions of the treatment group using the predefined treatment effect of 0, 4.4, 11, 17.6, and 22.

Figure 1 presents example distributions of five DGMs for three different levels using scores generated from the same latent Normal distribution, with the observed average treatment effect and SDs marked in brackets. The example dataset used a sample size of 400 since it is the closest to the median sample size of the 114 identified trials in our review.^3^

Table 4 shows the predefined and the observed means of the simulated PRO scores in the control and treatment groups, and the observed group differences. Table 5 presents the mean estimates from three different statistical methods under the five DGMs for each level using all observed estimates. Estimates for these methods under each DGM tended to decrease with an increase in the number of possible discrete scores, except for Median. For example, under DGM 5 where the treatment effect is predefined as 22-point on the original latent Normally distributed PRO scale, the average treatment effect in MLR is 21.33 for level 4, 21.03 for level 10, and 20.97 for level 26. Except for the Median, non-convergence was not seen for the other included methods. Median produced around 0.1% missing values when analysing level 4.

**Table 4.** Comparison of predefined and observed means and group differences of PRO scores in the control and treatment groups.

					Observed means								
		Predefined ‘truth’			Level 4			Level 10			Level 26		
Sample size	DGM	Control	Treat	x	Control	Treat	$\bar{x}$	Control	Treat	$\bar{x}$	Control	Treat	$\bar{x}$
100	1	50	50	0	49.93	49.98	0.05	49.98	50.05	0.07	49.93	49.98	0.05
200					49.95	50.04	0.08	50.01	50.06	0.05	49.96	50.00	0.05
400					49.99	50.01	0.01	50.03	50.05	0.01	49.98	50.00	0.02
800					50.01	50.01	−0.01	50.06	50.05	−0.01	50.01	50.00	−0.02
1200					50.01	49.99	−0.02	50.04	50.04	0.00	50.00	50.00	0.00
1600					50.02	50.01	−0.01	50.06	50.05	0.00	50.00	50.01	0.00
100	2	50	54.4	4.4	49.93	54.37	4.44	49.98	54.34	4.36	49.93	54.28	4.35
200					49.95	54.37	4.42	50.01	54.36	4.35	49.96	54.30	4.34
400					49.99	54.35	4.36	50.03	54.36	4.33	49.98	54.29	4.31
800					50.01	54.35	4.34	50.06	54.36	4.29	50.01	54.29	4.28
1200					50.01	54.34	4.34	50.04	54.35	4.31	50.00	54.29	4.30
1600					50.02	54.36	4.34	50.06	54.37	4.31	50.00	54.30	4.30
100	3	50	61	11	49.93	60.85	10.92	49.98	60.77	10.79	49.93	60.68	10.74
200					49.95	60.82	10.86	50.01	60.78	10.77	49.96	60.69	10.73
400					49.99	60.82	10.82	50.03	60.76	10.73	49.98	60.68	10.70
800					50.01	60.83	10.82	50.06	60.76	10.70	50.01	60.68	10.67
1200					50.01	60.84	10.83	50.04	60.76	10.72	50.00	60.68	10.69
1600					50.02	60.83	10.81	50.06	60.77	10.71	50.00	60.69	10.69
100	4	50	67.8	17.8	49.93	67.18	17.25	49.98	67.01	17.04	49.93	66.92	16.99
200					49.95	67.17	17.22	50.01	67.03	17.03	49.96	66.93	16.97
400					49.99	67.18	17.18	50.03	67.02	16.99	49.98	66.92	16.94
800					50.01	67.18	17.17	50.06	67.02	16.96	50.01	66.92	16.91
1200					50.01	67.19	17.19	50.04	67.02	16.98	50.00	66.92	16.92
1600					50.02	67.20	17.18	50.06	67.03	16.97	50.00	66.93	16.93
100	5	50	72	22	49.93	71.32	21.39	49.98	71.07	21.09	49.93	70.95	21.01
200					49.95	71.32	21.37	50.01	71.06	21.06	49.96	70.95	20.99
400					49.99	71.31	21.32	50.03	71.05	21.02	49.98	70.95	20.97
800					50.01	71.30	21.29	50.06	71.05	20.99	50.01	70.95	20.93
1200					50.01	71.31	21.30	50.04	71.06	21.01	50.00	70.94	20.95
1600					50.02	71.32	21.30	50.06	71.06	21.01	50.00	70.96	20.95

**Table 5.** Estimates of treatment effect from three statistical methods under five DGMs for three different levels.

					Estimated treatment effects								
		Predefined ‘truth’			Level 4			Level 10			Level 26		
Sample size	DGM	Control	Treat	x	MLR	Tobit	Median	MLR	Tobit	Median	MLR	Tobit	Median
100	1	50	50	0	0.050	0.041	0.155	0.069	0.059	0.164	0.048	0.042	0.066
200					0.082	0.099	0.573	0.050	0.051	0.202	0.047	0.044	0.099
400					0.013	0.018	−0.133	0.013	0.013	−0.020	0.020	0.021	−0.041
800					−0.007	−0.011	0.320	−0.015	−0.017	0.116	−0.017	−0.018	0.046
1200					−0.016	−0.016	0.147	−0.001	−0.002	0.031	0.000	0.000	0.000
1600					−0.008	−0.012	0.346	−0.003	−0.004	0.085	0.004	0.005	0.026
100	2	50	54.4	4.4	4.443	5.083	13.037	4.362	4.549	4.889	4.348	4.477	4.461
200					4.419	5.078	15.245	4.353	4.553	5.268	4.344	4.475	4.481
400					4.359	4.997	15.704	4.326	4.521	5.333	4.314	4.448	4.370
800					4.339	4.966	16.297	4.292	4.484	5.488	4.278	4.409	4.492
1200					4.337	4.970	16.077	4.308	4.499	5.394	4.295	4.425	4.534
1600					4.343	4.969	15.831	4.312	4.506	5.313	4.299	4.431	4.624
100	3	50	61	11	10.925	12.730	16.593	10.790	11.359	11.102	10.742	11.132	11.040
200					10.863	12.664	16.597	10.770	11.351	11.117	10.733	11.131	11.042
400					10.822	12.600	16.084	10.727	11.296	10.727	10.705	11.098	10.946
800					10.817	12.582	16.304	10.699	11.265	10.867	10.669	11.061	10.849
1200					10.831	12.604	16.077	10.717	11.282	10.700	10.685	11.075	10.666
1600					10.811	12.575	15.831	10.713	11.278	10.659	10.689	11.079	10.570
100	4	50	67.8	17.8	17.252	20.706	16.623	17.037	18.208	17.429	16.989	17.820	17.543
200					17.220	20.655	16.597	17.025	18.207	17.149	16.973	17.807	17.579
400					17.183	20.600	16.084	16.991	18.155	16.652	16.944	17.768	17.551
800					17.166	20.555	16.304	16.957	18.115	16.592	16.908	17.730	17.682
1200					17.187	20.583	16.077	16.976	18.137	16.494	16.924	17.745	17.717
1600					17.182	20.573	15.831	16.975	18.133	16.413	16.928	17.748	17.790
100	5	50	72	22	21.388	26.370	16.633	21.091	22.868	21.874	21.013	22.294	21.895
200					21.367	26.318	16.597	21.058	22.830	21.842	20.993	22.274	21.921
400					21.317	26.247	16.084	21.020	22.776	21.441	20.969	22.236	21.920
800					21.288	26.178	16.304	20.991	22.744	21.514	20.932	22.195	22.006
1200					21.299	26.190	16.077	21.011	22.766	21.298	20.947	22.213	21.949
1600					21.304	26.196	15.831	21.006	22.760	21.186	20.953	22.216	21.967

Each cell contains up to a maximum of 5000 estimates, with a total of 27,000,000 estimates. DGM: data-generating mechanism; Median: median regression; MLR: multiple linear regression; Tobit: Tobit regression.

### 3.2 Exploratory analysis

**Figure 2.**
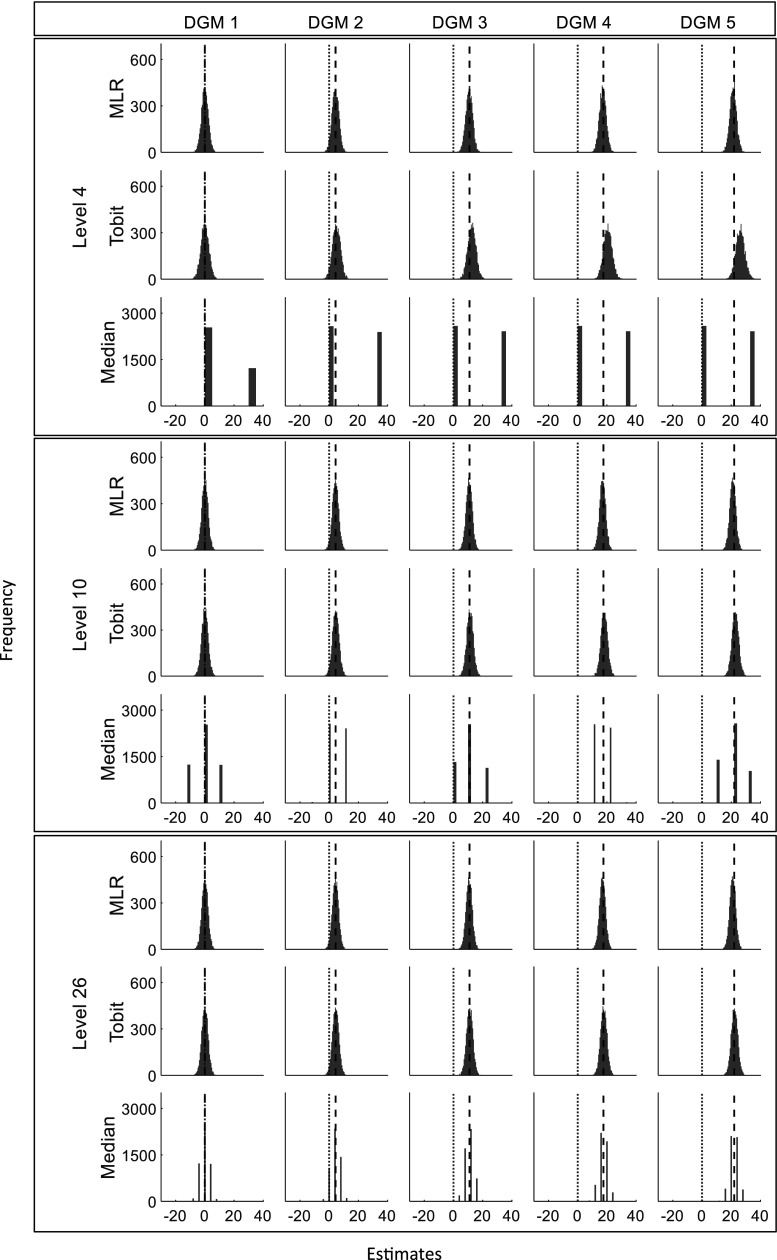
Histograms of estimates from three statistical methods for three different levels (sample size = 400). DGM: data-generating mechanism; Median: median regression; MLR: multiple linear regression; Tobit: Tobit regression. Note that different scales for the *y*-axis are used for MLR/Tobit and Median. The vertical dotted line represents estimates of 0 and the dashed line represents the predefined ‘truth’ of 0, 4.4, 11, 17.6, and 22 under five DGMs.

**Figure 3.**
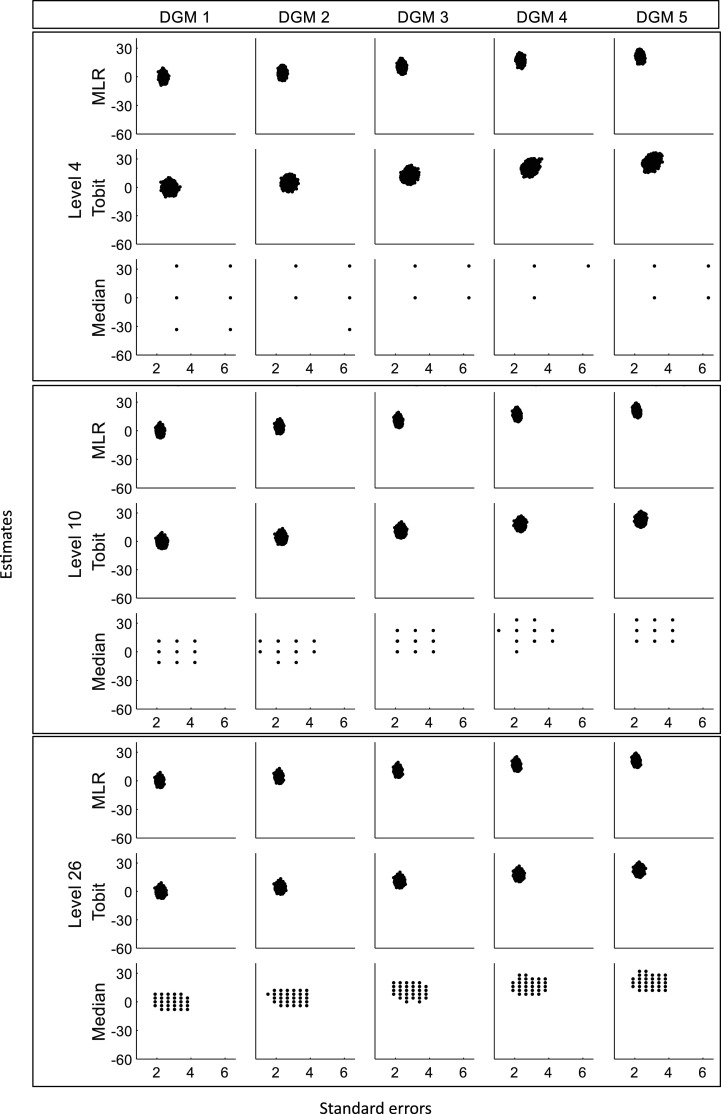
Scatterplots of estimates against standard errors for three different levels (sample size = 400). DGM: data-generating mechanism; Median: median regression; MLR: multiple linear regression; Tobit: Tobit regression.

**Figure 4.**
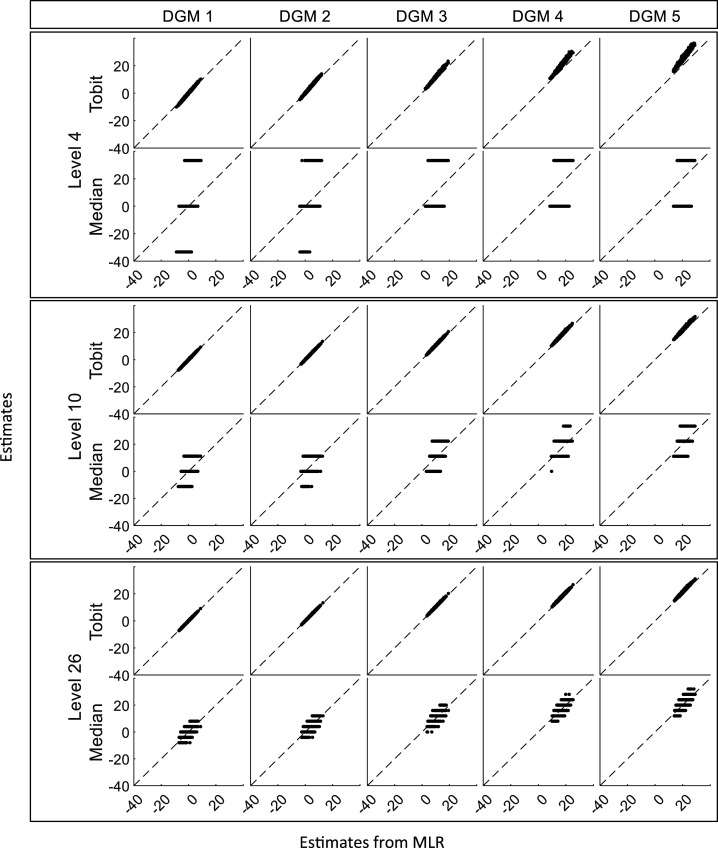
Scatterplots of estimates from Tobit and Median against estimates from MLR for three different levels (sample size = 400). DGM: data-generating mechanism; Median: median regression; MLR: multiple linear regression; Tobit: Tobit regression.

Figure 2 presents the distributions of the estimated treatment effects ( ${\hat{x}}_{i}$ ) produced from the three statistical methods under five DGMs for each level, where the dotted line represents estimates of 0 and the dashed line represents the predefined treatment difference of 0, 4.4, 11, 17.6, and 22 under five DGMs. Median only produced estimates at certain values that are around multipliers of the gap between two nearby PRO discrete values. For example, estimates from Median were around multiples of 11 for level 10. Figure 3 shows that no bivariate outliers were seen in the scatterplots of estimated treatment effects $\left({\hat{x}}_{i}\right)$ against their associated SEs $\left(\hat{SE}({\hat{x}}_{i})\right)$ by different statistical methods under the five DGMs. MLR and Tobit presented no relationship or a positive relationship between $\hat{x}$ and $\hat{SE}(\hat{x})$ , whereas the plots for Median had a stripped pattern. Figure 4 shows that the estimates from Tobit have good consistency with MLR in most cases, except when the magnitude of the predefined ‘truth’ increased, Tobit tended to produce numerically larger estimates than MLR when the possible discrete value was small, while estimates from Median keeps showing a stripped pattern in comparison to MLR.

### 3.3 Analysis of performance measures

The key performance measures include the bias that measures the accuracy of these statistical methods for estimating the true treatment effect, the coverage of 95% CI for including the true treatment effect, MSE, EmpSE, and the power or Type I error that measures the precision or the robustness of these methods.

**Figure 5.**
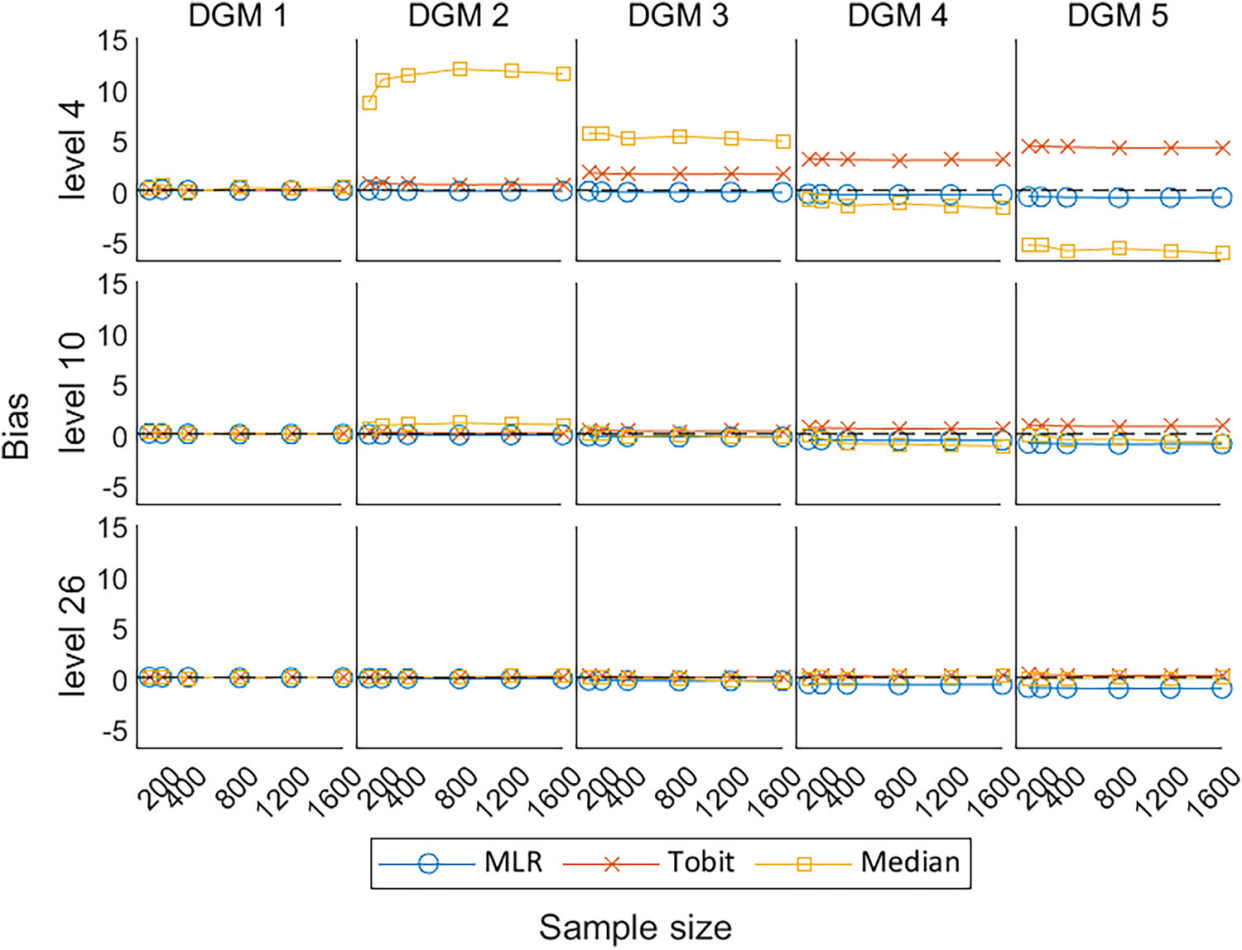
Line plots of bias for the three methods with the change of sample sizes for three different levels. DGM: data-generating mechanism; Median: median regression; MLR: multiple linear regression; Tobit: Tobit regression.

Figure 5 presents the change in bias of these three methods under the null hypothesis (DGM 1) and the alternative hypothesis (DGM 2–5). When the predefined ‘truth’ is zero, these methods were able to produce estimates close to the predefined ‘truth’. Their estimates fluctuated and gradually converged to the dashed line (bias = 0) with the increase in sample sizes. Median presented a larger bias than other methods, especially for PROs with a small number of levels. When analysing a small number of levels, especially level 4, Tobit tended to overestimate the treatment effect, and MLR tended to underestimate the treatment effect, but Tobit was more biased than MLR. However, when analysing a higher number of levels, the bias from Tobit became smaller than MLR.

**Figure 6.**
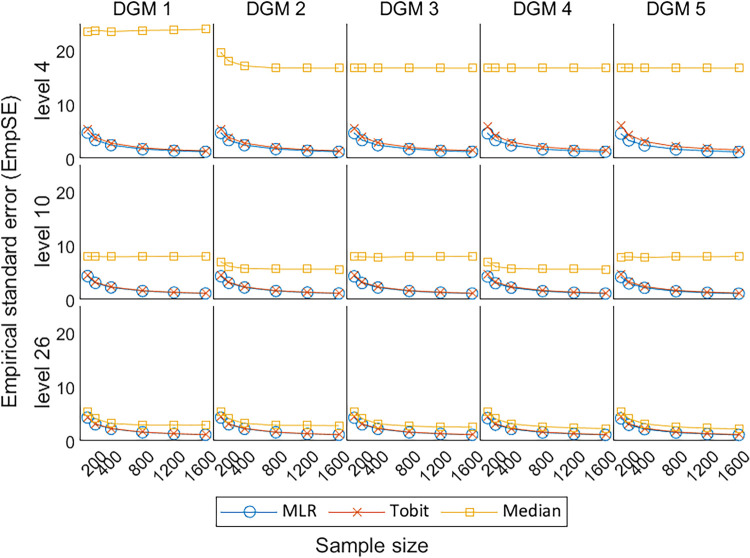
Line plots of EmpSE for the three methods with the change of sample sizes for three different levels. EmpSE: empirical standard error; DGM: data-generating mechanism; Median: median regression; MLR: multiple linear regression; Tobit: Tobit regression.

As shown in Figure 6, the EmpSE for MLR and Tobit remained similar under different scenarios, except that Tobit was associated with slightly higher EmpSE, i.e., less precision to the average estimates, when the number of levels is small. This indicates that Tobit was less precise than MLR for PROs with a small number of levels (i.e. level 4). Median tended to have less EmpSE and converge to the trend for MLR and Tobit, when analysing PROs with more number of discrete values (i.e. levels 10 and 26). Although the EmpSE of Median dropped dramatically with the increase in the number of levels, Median had the worst precision in comparison with MLR, and Tobit in all scenarios.

**Figure 7.**
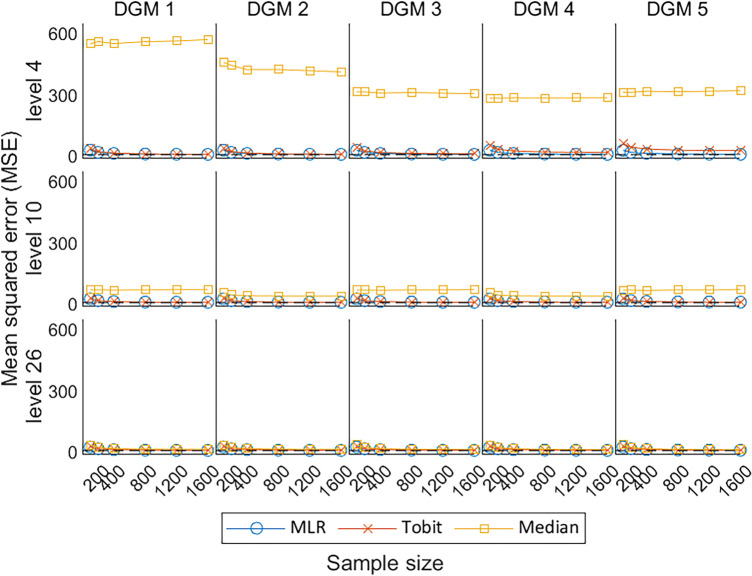
Line plots of MSE for the three methods with the change of sample sizes for three different levels. MSE: mean squared error; DGM: data-generating mechanism; Median: median regression; MLR: multiple linear regression; Tobit: Tobit regression.

Under the null hypothesis, the MSE estimated by the MLR and Tobit are similar to each other, decreasing with the increase in sample size, and converges to the dashed line representing MSE = 0; whereas Median presents comparatively high MSE (Figure 7). Under the alternative hypotheses, MLR and Tobit show similar trends, with Tobit having a slightly larger MSE for level 4 but a smaller MSE for level 26 when the predefined ‘truth’ was large (i.e. under DGM 4 and 5). This indicates that Tobit was less precise than MLR for a small number of levels (i.e. level 4), but more precise than MLR for a large number of levels (i.e. level 26). Despite the MSE of Median decreasing dramatically with the increase in the number of levels, Median had the worst precision in comparison with MLR and Tobit in all scenarios.

**Figure 8.**
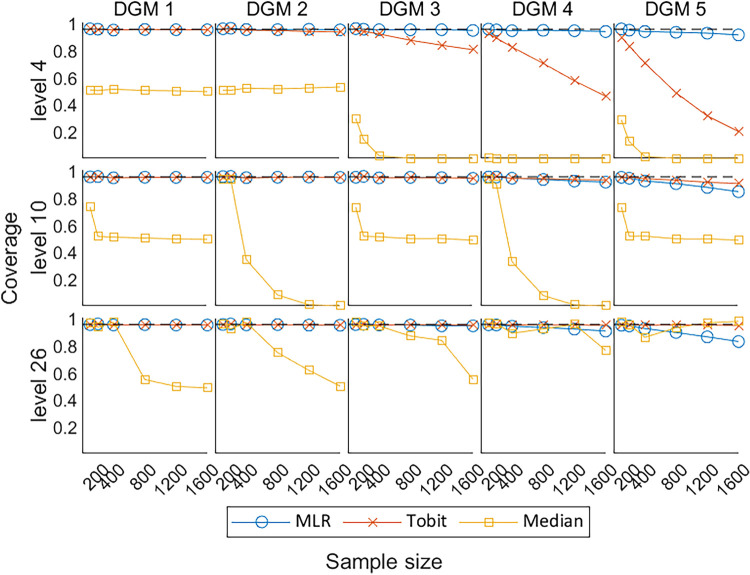
Line plots of coverage of 95% CIs with the change of sample sizes for three different levels. CIs: confidence intervals; DGM: data-generating mechanism; Median: median regression; MLR: multiple linear regression; Tobit: Tobit regression.

Figure 8 shows the coverage of 95% CI for ${\hat{x}}_{i}$ with the change in sample size, where the dashed line represents coverage = 0.95. Under the null hypothesis (DGM 1), most methods were able to produce the estimates of zero, except for the Median. Under the alternative hypothesis (DGM 2–5), the coverage of MLR and Tobit deviated from the reference line with the increase in the predefined ‘truth’ (i.e. the predefined treatment effect) and the number of observations (i.e. sample sizes) and this phenomenon was more obvious for Tobit at level 4. However, for PROs with a larger number of levels (i.e. for levels 10 and 26), Tobit had less deviations from the reference than MLR did. Median had better performance in coverage when the simulated PRO scores became more continuous (level 26), but its coverage remained no better than MLR and Tobit under most scenarios.

**Figure 9.**
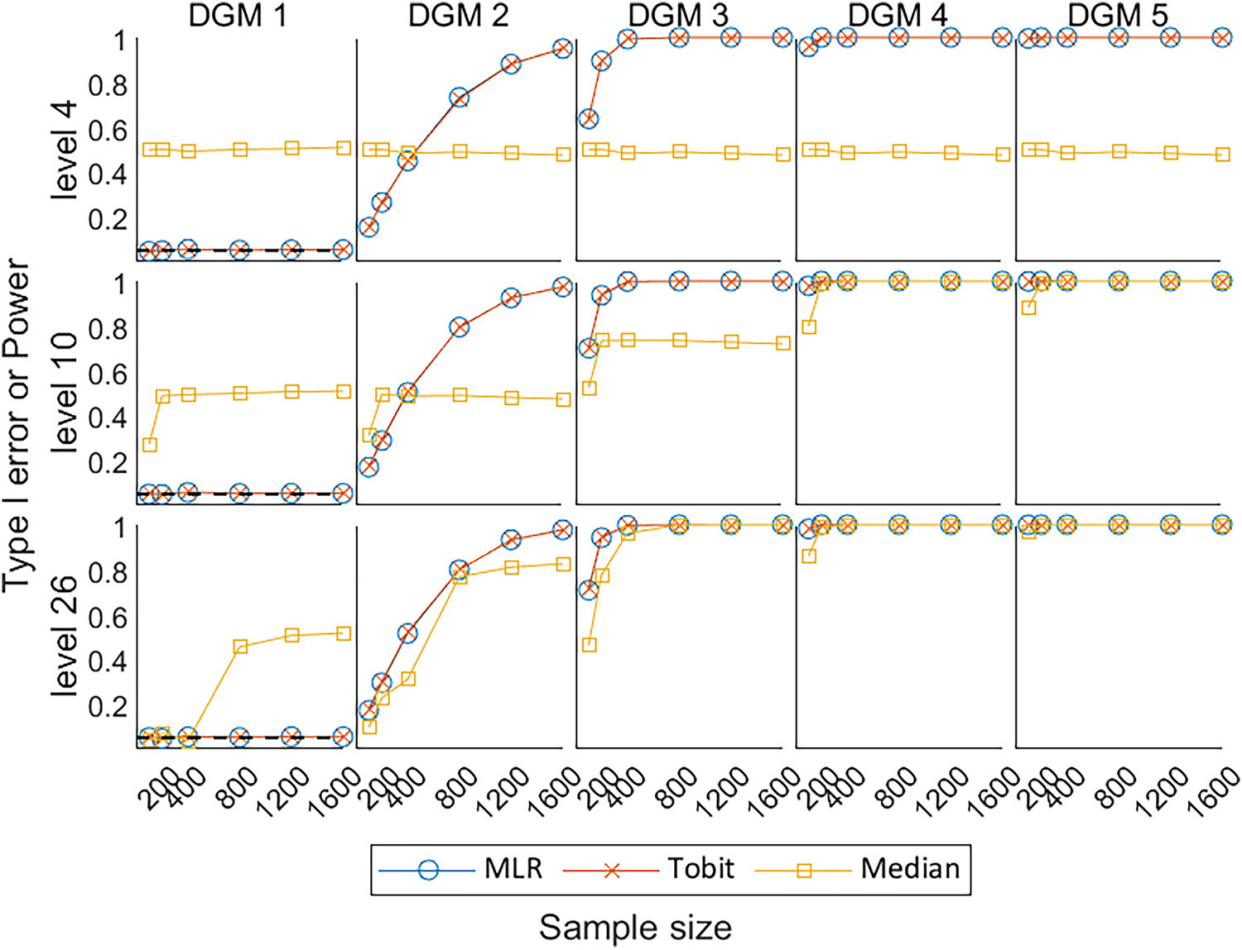
Line plots of Type I error under the null hypothesis (DGM 1) and power under the alternative hypothesis (DGM 2–5) with the change of sample sizes for three different levels. DGM: data-generating mechanism; Median: median regression; MLR: multiple linear regression; Tobit: Tobit regression.

Figure 9 shows the Type I error under the null hypothesis of no treatment effect (DGM 1) and power under the alternative hypothesis of a variety of predefined non-zero treatment effects (DGM 2–5) for ${\hat{x}}_{i}$ with the change in sample size, where the dashed line represents the Type I error of 0.05 under DGM 1. The lines of MLR and Tobit overlapped with each other at *y* = 0.05 when the significance level was set at α=0.05 . The Median had a Type I error at around 0.5 under DGM 1, indicating that Median produced far more false positives than MLR and Tobit. Under DGM 2–5, the increasing trend in power of MLR and Tobit overlapped with each other, while the power of Median was lower than MLR and Tobit under all DGMs for level 4 and under DGM 2–5 for levels 10 and 26, indicating Median is less likely to correctly reject the null hypothesis than MLR and Tobit under most scenarios.

## 4 Discussion

This article compared the model performances of three statistical methods (i.e. MLR, Tobit, and Median) in estimating the predefined treatment effects of PROs with different number of discrete values (i.e. levels 4, 10, and 26) under a range of scenarios in RCT settings, using Monte Carlo simulation methods.

The key performance measures of the three statistical methods were compared, i.e. bias, coverage, EmpSE, MSE, Type I error, and power. MLR performed better than other methods when analysing simulated PRO datasets in RCT settings with a wide range of possible dimension levels. It was associated with little bias in the estimate, small EmpSE, and appropriate coverage of 95% CI compared to Median and Tobit under most scenarios. Tobit had a slightly smaller bias with its coverage of 95% CI closer to the 0.95 reference line than MLR when the predefined treatment effect was large (i.e. DGM 5) for levels 10 and 26. However, it had a larger bias and worse coverage than MLR when the number of possible discrete values was small (i.e. level 4). Median showed extremely large bias and errors, associated with low power and coverage compared to other statistical methods for most scenarios, especially under level 4.

MLR is recommended as the universal statistical method for the analysis of latent continuous PRO dimension scores with equally spaced scoring in balanced two-arm RCT settings. This recommendation is a trade-off on various aspects of the model performance in the simulation analysis and technical details of these methods, and we believe that the same statistical method is preferred for the analysis of all dimension scores in a multidimensional PRO such as SF-36 if applicable. MLR is also recommended by other studies that compared different sets of statistical methods for PRO analysis.^20,33,34^ From a medical statistician's point of view, MLR requires no transformation of the response variable, it produces point estimates that are based on the untransformed scale of measurement and are easy to interpret, and is a robust method when faced with the violation of model assumptions,^35,36^ particularly when the population mean and difference in population means between the randomised groups is an appropriate population-level summary measure of the treatment effect. From a health economist's point of view, the mean treatment effect in a PRO is commonly used for the calculation of incremental cost-effectiveness ratio, which represents the additional cost of one unit increase in a PRO to inform the results of a cost-effectiveness analysis, compared to other population-level summary measures such as medians or odds ratios.^37^

Tobit is recommended to analyse PROs when the treatment effect is believed to be very large and when the PRO is less discrete (i.e. at least 10 levels). Tobit is known to be consistent and efficient under the Normality assumption of residuals and homoscedasticity.^11,38^ When analysing a small number of levels, especially level 4, Tobit tended to overestimate the treatment effect, and MLR tended to underestimate the treatment effect, but Tobit produced more biased estimates than MLR. The undercoverage of Tobit under level 4 can result from bias, heteroscedasticity, or non-Normality.^20^ Tobit had better model performance for the analysis of PRO data with more possible values (i.e. levels 10 and 26), which is evident by previous studies.^11,38^ It is worth noting that the application of Tobit requires an additional premise that the PRO scores exceeding the lower and upper limits are believed to be meaningful.

Censored least absolute deviations (CLAD) regression that generates latent median estimates can be used as a substitute for Tobit regression when analysing a PRO with a ceiling effect. Austin (2002) compared Tobit, Median, and CLAD for analysing health utility index (HUI) scores^19^ in a large sample, of 14,460 subjects, from a non-randomised cross-sectional design, and found that Median and CLAD tend to produce estimates with similar patterns and their estimates tend to be shrunk to zero compared to MLR and Tobit regression. They found conflicting evidence of the effect of a binary outcome (gender) and the PRO and because this was an empirical study, the true effect (of gender) was unknown. Austin^19^ recommended the use of CLAD for its low predictive accuracy in practice and its robustness to heteroscedasticity and non-Normality of errors theory. This differs from our recommendations, as our focus is to generate simulated datasets of continuous latent PROs with equally spaced scoring to compare statistical methods that generate means or medians as the population-level summary measure for the purpose of estimating robust and reliable treatment effect estimates instead of making predictions. In an empirical analysis comparing several statistical methods (including CLAD) for the analysis of PROs using three RCTs, we found that the CLAD, as a censored form of Median, was not computationally efficient and took extra time to run compared to other methods. This is because it requires compulsory bootstrapping techniques to estimate the standard errors and hence an additional stage to calculate the *p*-values and CIs, which would complicate the simulation with an extra layer of resampling.

Although Median, a non-parametric statistical method, theoretically makes no assumption about the distributions of the outcome variable, it has been found to fail when the outcome variable is discrete.^39^ Some degree of smoothness should be artificially imposed to apply quantile regression to ordered data, such as adding uniformly distributed noise to the ordered data.^40^ This explains why the simple median regression produced unsatisfactory estimates in striped patterns with poor performance when analysing the small number of possible levels in the empirical analysis and the simulation analysis.

Previous studies^11,19,20,23^ have compared various statistical methods for the analysis of different types of PROs, but these studies focused on various groups of methods, proposed different DGMs, and made inconsistent recommendations on what statistical methods are more appropriate to use.

We agree with Bottomley et al.^41^ that establishing predefined criteria to assess statistical methods used in PRO analysis is crucial for making scientifically informed choices. The choice of statistical methods for analysing PROs depends on multiple factors, including the nature of the outcome variable, the adherence of the data to the method assumptions, and the criteria set for method evaluation by different stakeholders. The study design and the research question are the fundamental factors and can influence the selection of a statistical method, for example, whether the PRO is believed to be measuring an underlying latent variable, and whether the underlying latent variable is believed to be fundamentally continuous or discrete; whether the study design requires the adjustment for clustering, time effects, or unbalanced data; and whether the PRO analysis is to be used for making predictions, estimating a treatment effect, or measuring influencing factors. These factors vary study-by-study and need to be carefully considered when researchers are making the decision on what statistical methods to use.

This study compared three commonly used or proposed statistical methods for the analysis of PROs under multiple scenarios, with a thorough comparison of the performance measures of these included methods. The recommendation of this paper is based on the technical details of these methods, and their performance in the simulation analysis, with the aim of estimating the treatment effect of the latent continuous PROs with equally spaced discrete scores ranging from 0 to 100 under a two-arm balanced design RCT. The outcomes can be extrapolated to other popular PROs similar to SF-36 that focus on functions in the domains of health assessed, such as the European Organization for the Research and Treatment of Cancer Quality of Life Questionnaire (EORTC QLQ-C30),^42^ Beck Depression Inventory (BDI),^43^ and Hospital Anxiety and Depression Scale (HADS).^44^

However, the appropriateness of recommending MLR or Tobit to analyse extremely skewed distribution with ceiling effects needs to be investigated further. The extreme skewness and ceiling effects are commonly seen in preference-based PROs that typically apply weights based on patients’ preferences through attached algorithms to generate health utility scores such as Short Form 6-Dimensions (SF-6D),^45^ Health Utilities Index (HUI),^46^ and EuroQol-5 Dimension (EQ-5D).^47^ When the PRO scores are extremely skewed with ceiling effects, the proportions or odds ratios might be a better population-level summary measure of the treatment effect in RCTs, than differences in measures of central locations such as means or medians. In these circumstances, the use of MLR or Tobit that produces mean estimates may be flawed. Utilising the estimand framework, statistical methods such as the beta-binomial or ordinal regression have a different estimand (where the population-level summary measure is an odds ratio rather than a difference in means) to MLR and Tobit, and compare the outcomes between treatment groups in a different way. Methodological research has been published to deal with the analysis of health utility scores.^11,19–24^ We believe that it is interesting to discover to what extent of the skewness may alter the recommendation of using MLR to analyse PRO dimension scores, but this is outside the scope of this study.

This simulation study has the following limitations:

First, this simulation study considered 90 scenarios, i.e. five predefined treatment effects to produce PRO scores under three different numbers of levels (i.e. 4, 10, and 26), using six different sample sizes. These scenarios are not able to represent all possible distributions of PRO that would appear in an RCT setting. However, the selection of these parameters was evidence-based, and the same set of parameters were used to compare performance measures across PRO data with different levels.
Second, the dilemma of this study is finding the appropriate distribution with predefined parameters that can depict the distribution of SF-36 dimension scores, which is typically bounded, skewed, and discrete. The use of Normal distribution assuming an underlying latent variable to generate simulated datasets may favour MLR and Tobit.^25^ Alternatively, other distributions could be used to produce the simulated datasets,^23,48^ for example, the use of beta-binomial distribution to generate PRO data is seen in the simulation analysis comparing two approaches to achieve the beta-binomial regression by Najera-Zuloaga et al.,^48^ but potentially it would favour the beta-binomial regression instead and produce an odds ratio as the population-level summary measure. This is outside the scope of our study. The Normal distribution is preferred to other distributions in this simulation analysis not only due to its simplicity and extensive applicability,^49^ but also because we believe that the PRO is inherently continuous with no boundaries.
Third, for the treatment effect we assumed a simple ‘location shift’ on the underlying latent continuous normally distributed PRO, i.e. the underlying distribution for the outcome is ‘shifted’ to the right so the mean of the new distribution is 4.4, 11, 17.8, or 22-points higher with the mean and the SD of the Normal distribution was set fixed for all scenarios. Again, this assumption may favour statistical methods that assume the outcome is continuous. If the mean was set at a higher value, the location shift would be more influenced by the ceiling effect given the current set of predefined treatment effects, i.e. there would be more values censored at the upper bound, and thus setting the mean at a higher value will make the observed treatment effects farther from the predefined value. If the mean was set at a lower value, there would be more space to move up on the scale, such that the location shift would be less influenced by the ceiling effect given the current set of predefined treatment effects, i.e. the observed treatment effects would be closer to the predefined treatment effects.
In addition to the ‘location shift’, there could also be a change in the shape of the distribution. For example, when simulating from Normal distributions, the SD could be different in the two groups due to the effect of treatment. However, this simulation study only considered the simple scenario where the SD of two groups was assumed the same. The discretisation procedure is the only factor that may change the shape of the simulated PROs in this simulation besides the ceiling effect.
Fourth, discretisation techniques were required in this study to simulate the discrete characteristics of the PRO data. The simulated datasets in this analysis were discretised into equally spaced values, such that whether the conclusion from this simulation is generalisable to non-equally spaced PROs needs to be further investigated. However, there is no standard way to discretise the PRO data. Also, the discretisation of the Normally distributed latent PRO into 4, 10, or 26 levels or discrete values may mean that all the statistical methods produce a slightly biased estimate of the true treatment effect as the observed differences in mean scores from the simulations show. Therefore, this discretisation procedure makes it impossible to observe the exact predefined treatment effect of 4.4, 11, 17.8, and 22 points on the underlying Normally distributed scale, except when the predefined treatment effect is set at zero under the null hypothesis.

Furthermore, this study considered a limited number of statistical methods that produce means or medians and excludes methods that produce estimates of log odds ratio such as ordinal logistic regression or beta-binomial regression. This is because the population-level summary measures of these methods are by nature different, for example, MLR produces estimates of differences in means while beta-binomial regression produces estimates in odds ratio, and strictly speaking, these methods with different population-level summary measures are not comparable. As the focus of this study is to compare statistical methods for the analysis of latent continuous PROs, with the target population-level summary measure of central tendency i.e. means or medians, some statistical methods are naturally excluded as outside the scope of our study, and the model performance of these methods for analysing PROs need to be further explored.

This article focused on a simple situation where there is a single baseline and a single post-randomisation assessment of an outcome, and compared the statistical methods that are suitable for such an analysis. Other factors that can affect the accuracy and robustness of the analysis of PRO scores, such as measurement error or missing data, are not considered. For future research, there exists an opportunity to explore statistical methods for correlated responses, strategies to deal with missing values, and techniques to estimate the clustering effect for the analysis of PROs. In addition, other distributions such as the beta distribution or beta-binomial distribution can be considered to generate the simulated dataset to investigate whether the model performance of each statistical method may change.

## 5 Conclusions

This article compared three common statistical methods for the analysis of PRO data using Monte Carlo methods in various scenarios. Considering a single baseline and a single post-randomisation assessment of an outcome, MLR shows better performance than Tobit and Median in most scenarios, and we suggest using it as the universal statistical method for the analysis of latent continuous PROs with equally spaced scoring in balanced two-arm RCT settings, especially when one statistical method is preferred to analyse multiple dimension scores, and Tobit is recommended as an alternative method if the treatment effect is believed very large. Future work involves the exploration of statistical methods for analysing PROs in more complex scenarios.
